# Chemical Profile of Turnip According to the Plant Part and the Cultivar: A Multivariate Approach

**DOI:** 10.3390/foods12173195

**Published:** 2023-08-24

**Authors:** Jing Yang, Jiashu Lou, Weiwei Zhong, Yaochen Li, Yong He, Shiwen Su, Xianzhi Chen, Biao Zhu

**Affiliations:** 1 Key Laboratory of Quality and Safety Control for Subtropical Fruit and Vegetable, Ministry of Agriculture and Rural Affairs, Collaborative Innovation Center for Efficient and Green Production of Agriculture in Mountainous Areas of Zhejiang Province, College of Horticulture Science, Zhejiang A&F University, Hangzhou 311300, Chinaliyaochen@stu.zafu.edu.cn (Y.L.); heyong@zafu.edu.cn (Y.H.); 2 Wenzhou Academy of Agricultural Sciences, Wenzhou 325006, Chinachenxianzhi@126.com (X.C.)

**Keywords:** *Brassica rapa*, chemical assessment, nutritional composition, biocompounds, elemental composition

## Abstract

Turnip (*Brassica rapa* subsp. *rapa*) is a cruciferous plant cultivated worldwide that serves as a source of nutrients and bioactive compounds. Most turnip studies have focused on a few compounds or on part of the plant. The establishment of a complete chemical profile of different plant parts would facilitate its use for nutritional and medicinal purposes. In the current study, mineral elements, soluble sugars, free amino acids (FAA), total phenols (TP), total flavonoids (TF), and glucosinolates (GS) were quantified in the leaves, stems, and roots. Results were compared for 20 strains of turnip. The outcomes showed significant differences between parts of the plant and strains. The leaves exhibited the highest TF, TP, indispensable FAA, and microelement levels, and they showed a higher GS. Moreover, the stems had a high content of GS and macroelements. Furthermore, the roots showed high levels of free sugars and total FAA. The findings of this work provide the basis for utilizing each part of the turnip plant based on its chemical composition.

## 1. Introduction

The turnip is a plant in the *Brassica* genus (formerly known as the family *Cruciferae*) that could grow in high-altitude conditions, and it is cultivated worldwide because of its economic importance. The root of the turnip, which is the main edible part, can be eaten raw (as in Europe, Asia, America, and North Africa), fermented, pickled, boiled, or cooked [1,2]. The turnip leaf is also used as a culinary ingredient or potherb [1]. In addition, turnip could be used as fish food, as fodder for livestock feeding, as a substitute resource for the production of biodiesel, or for use in biofumigation due to its high content of glucosinolates and gaseous by-products [1,2]. Furthermore, turnip could be processed into micron powder and turnip beverages, and it could be used for microencapsulation, among other uses [3,4]. Turnip is well documented for its nutritive value, which is a source of carbohydrates, vitamins, elements, and other nutrients, as well as for its medicinal properties. Turnip was also documented as a traditional therapeutic agent (e.g., for liver and kidney diseases or flu); it is thought to have anti-cardiovascular disease, antitumor, antioxidant, and anti-inflammatory effects; and it can combat various other ailments, which are associated with different groups of novel phytochemicals, including glucosinolate (GS), polyphenols, and flavonoids; these levels are higher than those in other important root vegetable crops, such as radish [1,5,6].

Glucosinolates (GSs) are a group of secondary metabolites mainly found in cruciferous vegetables, such as turnip, broccoli, cabbage, and kale [7]; these play a crucial role in the prevention and control of plant diseases and pests, they regulate the flavor of vegetables, and most of them are thought to be beneficial to human health (i.e., with anti-cancer properties) [8,9]. Turnip contains a considerable amount of GSs. In fact, the total GS content in turnip greens was higher than that in kale (*B. oleracea* var. *acephala*), and it is comparable to Ethiopian mustard (*B. carinata* Braun) [1,8].

Polyphenols, including flavonoids, are found throughout the different parts of the turnip plant. These compounds exhibit antioxidant activity against reactive oxygen species. Furthermore, polyphenols potentially influence human health by reducing the risk of myocardial infarction and diabetes, as well as improving lipid profiles, blood pressure, and insulin resistance [5,6,10].

Free amino acids can be directly absorbed by the human body, which not only maintains the metabolism, immunity, and growth of organisms, but could also effectively scavenge free radicals as antioxidants [11]. Sweetness is influenced by soluble sugars [12], and the taste of horticultural crops is directly affected by the soluble sugar content, type, and composition ratio. Mineral elements are essential for plant growth, and the human body also needs to take in a variety of elements from food to maintain a good level of health [13]. The content of calcium, magnesium, and potassium in turnip is generally 1–3 times higher than that in cabbage, cauliflower, kale, and other cruciferous vegetables [14]. These elements are interrelated, and they influence the nutritional value of vegetables. In addition, technological processing affects the contents of turnip.

The flavor and nutritional quality of vegetables are the key factors affecting consumers. Some studies have been previously conducted that consider the turnip’s nutritional value, mainly as a root vegetable; however, fewer specific and detailed research papers systematically focus on the nutritional profile of different parts of different turnip species.

This study hypothesizes that determining the chemical composition of turnip parts can support the appropriate use of plants for nutritional and medicinal purposes. Therefore, the objectives of this research were as follows: (i) to assess the nutrient and biocompound contents in the roots, leaves, and stems of the turnip plant; and (ii) to establish the chemical profile of turnip strains (traditional and hybrid cultivars) using different multivariate methods.

## 2. Materials and Methods

### 2.1. Plant Material

Twenty strains of turnip (*Brassica rapa* subsp. *rapa*) plants were provided by the Wenzhou Academy of Agricultural Sciences at the commercial maturity stage. Nine strains were locally used traditional cultivars and eleven strains were hybrid; the details of these strains are cross-listed in Table S1.

All of the plants were cleaned with soft towels and divided into root (R), stem (S), and leaf (L), and then they were immediately frozen in liquid nitrogen. The samples were ground into powders using a grinding mill (AQ-180E, Nailappliance, Ningbo, China), and stored at −20 °C for analysis after freeze-drying in a vacuum freeze dryer (GAMMA 1-16 LSC, Martin Christ) for 72 h.

### 2.2. Analysis of Mineral Elements

The mineral elements were quantified, as described by Ruan et al. [15]. The freeze-dried powder (0.50 g) was digested with concentrated HNO_3_ (10 mol/L), fixed with water to 50 mL, and analyzed for minerals with a full-spectrum direct-reading Inductively Coupled Plasma–Optical Emission Spectrometer (ICP-OES) (Thermo Jarrell Ash, IRIS/AP) with ThermoSpec/CID V2.10.09.

### 2.3. Analysis of Soluble Sugar

The soluble sugar was extracted and measured according to the method of Kowalska et al. [16] with some modifications. The extracts were prepared using 0.10 g of freeze-dried powder and 6 mL of distilled water, and extracted in a water bath at 65 °C for 20 min. The extraction was then filtered by a 0.45 μm microporous filter prior to high-performance liquid chromatography (HPLC) analysis.

A Waters sugar-Pak I column WAT084038 (6.5 × 300 mm, 10 μm) was used for sugar separation, and HPLC (Agilent1200, Santa Clara, CA, USA) with a differential refractive index detector (G1362A, Agilent, Santa Clara, CA, USA) and Agilent ChemStation software were used for sugar identification and quantification. A 5 μL aliquot of each sugar sample was injected, and they were eluted with ultrapure water at a flow rate of 0.8 mL/min. The temperature of the column was 85 °C. The contents of glucose, sucrose, and fructose in the samples were calculated according to the standard curves drawn from the results of the injection.

### 2.4. Analysis of Free Amino Acids

The free amino acids were determined according to a previously described study [17]. The extracts were prepared using 0.1000 g of freeze-dried powder and 2.5 mL of ultrapure water. The samples were extracted in an ultrasonic bath at room temperature for 60 min. The extract was filtered through a 0.22 μm filter. A total of 10 μL of a sample solution, 70 μL of AccQ buffer, and 20 μL of derivatives (Waters AccQ Tag^TM^ reagent kit for amino acids analysis) were mixed for 15 s and heated in an oven at 55 °C for 10 min. The samples were then analyzed using the AccQ Tag system (Waters Corporation, Milford, CT, USA) by Waters Arc HPLC with a UV–visible light detector (2489) and Empower 3 Software, using an AccQ Tag^TM^ amino acid column (100 mm × 2.1 mm, 1.7 μm, Waters Corporation, Milford, CT, USA). The rate was 1 mL/min. Mobile phase A was the AccQ-Tag A solution diluted at 1:10 (*v*/*v*) with ultrapure water, mobile phase B was acetonitrile, and mobile phase C was ultrapure water. The content of each amino acid quantity in the sample was calculated from the standard curve drawn from the injection results.

### 2.5. Analysis of Total Polyphenols and Total Flavonoids

The total polyphenol (TP) and total flavonoid (TF) contents were determined as previously described [17].

The content of TP was measured using the Folin–Ciocalteu method [18]. The extracts were prepared using 0.1000 g of freeze-dried powder and 10 mL of 80% methanol. An aliquot (0.6 mL) of the extract, 3 mL of Folin–Ciocalteu reagent, and 2 mL of 7.5% Na_2_CO_3_ were mixed. The mixture was incubated for 60 min in the dark at room temperature, and the TP content was determined by the absorbance at 765 nm with a spectrophotometer (UV-2600, SHIMADZU, Tokyo, Japan). The content of total phenols is expressed as equivalent mg of gallic acid per gram of the dry sample.

The determination of TF was performed following the methodology proposed by the AlCl_3_ colorimetric method [19]. Additionally, 20 mg/mL of the extract was prepared using 60% ethanol. The extract of 2 mL was used with 0.4 mL of AlCl_3_ (10%, p/v) and 0.4 mL of 5% NaNO_2_. Subsequently, the mixture was vortexed and incubated for 6 min before 4 mL of NaOH (4%) was added to stop the reaction. Finally, the absorbance at 510 nm was measured using a spectrophotometer (UV-2600, SHIMADZU, Tokyo, Japan) using 60% ethanol as a blank. The total flavonoid content is expressed as the equivalent mg of rutin per gram of the dry sample.

### 2.6. Analysis of Glucosinolate Profiles

The glucosinolates (GSs) were determined as previously described with a slight modification [20]. In a 10 mL tube, 0.25 g of lyophilized powder was placed and extracted with 4 mL of 70% boiling methanol, using 200 μL of 3 mM glucotropaeolin (Sigma-Aldrich, St. Louis, MI, USA) as the internal standard, in a 75 °C water bath for 10 min. The solution was added with 1 mL of 0.4 mM barium acetate and then centrifuged at 8000 rpm for 10 min. The supernatant was transferred to a volumetric bottle and the residue was extracted twice with 3 mL of 70% boiling methanol. Three supernatants were combined and made up to a final volume of 10 mL with 70% methanol. A 5 mL aliquot was loaded onto a 1 mL mini-column (JT Baker, Phillipsburg, NJ, USA) containing 0.5 mL of activated DEAE SephadexTM A-25 (Amersham Biosciences, Uppsala, Sweden) and 200 μL of 2 mg/mL sulfatase (Sigma-Aldrich, St. Louis, MI, USA). The resultant desulfoglucosinolates were eluted with 5 mL of ultra-pure water and then filtered through a 0.22 μm filter.

The GS profile was analyzed using a Waters Acquity Arc UHPLC system (Waters Corporation, Milford, CT, USA) with Empower 3 Software, using a prontosil ODS2 column (250 mm × 4 mm, 5 μm, Bischoff, Leonberg, Germany). An aliquot (20 μL) of eluent was monitored with a UV–visible light detector (2489) set at 229 nm at 30 °C. The mobile phase was ultra-pure water (A) and acetonitrile (Tedia, Fairfield, CT, USA) (B) in a linear gradient from 0 to 20% B for 34 min, and then just constant 20% B for 6 min, followed by 100% B and 0% B prior to the injection of the next sample.

### 2.7. Statistical Analysis

The values of all indexes determined in the experiment were recorded in triplicate and the results are presented as the mean ± standard deviation (SD). The data were processed using the SPSS Software (Version 22.0. Armonk, NY, USA: IBM Corp.). One-way ANOVA and Duncan’s test were used to evaluate the significant differences (*p* < 0.05). Principal component analysis (PCA) was applied to evaluate the chemical compounds of 3 plant parts in 20 turnip strains, including GSs (except PRO), TF, TP, macroelements, microelements, soluble sugars, and indispensable amino acids. Factor analysis (FA) was performed to calculate the comprehensive chemical value scores of different parts of 20 turnip strains. In addition, correlation analysis and cluster analysis were used to detect to illuminate the difference and relevance of traits among genotypes and parts in turnips, as well as the correlation between various components of metabolites. Otherwise, hierarchical cluster analysis (HCA) was applied to classify all metabolites of 11 turnip strains containing kinship in 3 parts, and the relative distance between parents and offspring was further calculated. The results were plotted by Origin 2023 software (OriginLab.Corp., Northampton, PA, USA).

## 3. Results

### 3.1. Elemental Composition

**Figure 1:**
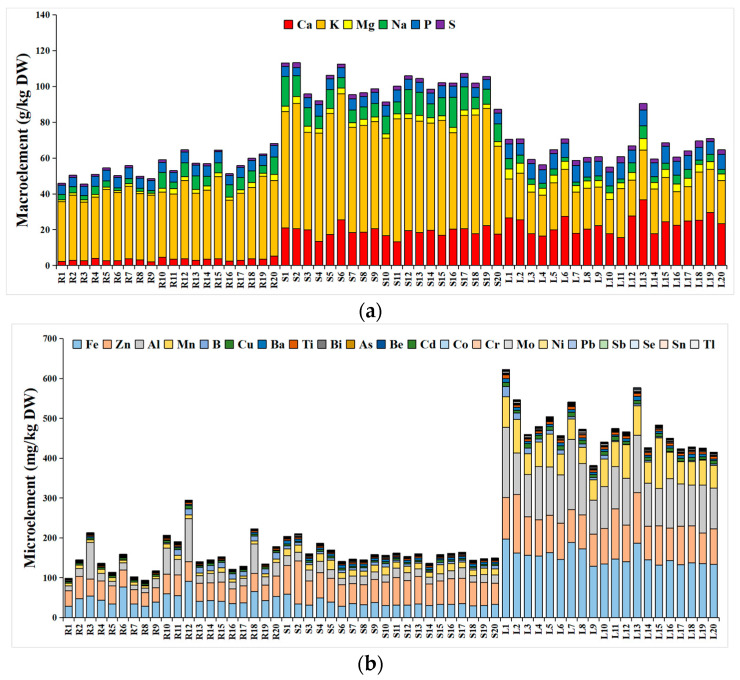

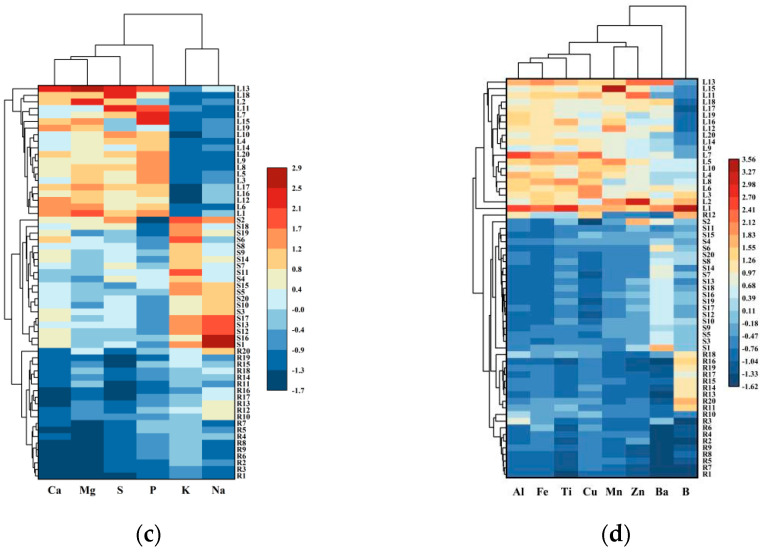
Distribution and cluster heat map of mineral elements in 3 plant parts of 20 turnip strains. (**a**) Content of macroelements; (**b**) content of microelement; (**c**) cluster heat map of 6 macroelements; and (**d**) cluster heat map of 8 microelements. (R: root; S: stem; L: leaf).

Six macroelements and twenty-two microelements detected in turnip are shown in Figure 1a,b. The total content of six macroelements, including Ca, K, Mg, Na, P, and S, ranged from 45.42 to 113.27 g/kg DW. The results showed the highest value of K content (18.72–70.40 g/kg DW) in all the parts of almost all 20 strains, and then P (4.51–9.57 g/kg DW) in the root and Ca (2.07–36.77 g/kg DW) in the stem and leaf. In general, the order of the average amount of each element slightly differed in different parts, namely K > P >Na > Ca > Mg > S in the root, K > Ca > Na > P > Mg > S in the stem, and K > Ca > P > Mg > Na > S in the leaf. Hence, the total content of six macroelements from the stem was higher than the root and leaf, owing to the high level of K, Ca, and Na (Figure 1c).

Considering their biological role in humans, the 22 microelements determined in this work are generally divided into four categories: (1) essential microelements, namely Fe, Zn, Mn, Se, Cu, Mo, and Co; (2) trace elements with beneficial effects, namely boron (B) and vanadium (V); (3) trace elements with toxic effects, namely Cr, Pb, Cd, and As; and (4) others, namely Al, Be, Ni, Sb, Tl, Sn, Bi, Ba, and Ti [21,22,23]. The average content of total microelements in the leaf (472.61 mg/kg DW) was evidently higher than that in the root (153.40 mg/kg DW) and stem (159.55 mg/kg DW). Among them, the total content of 8 maximum microelements (Fe, Zn, Al, Mn, B, Cu, Ba, and Ti) (Figure 1d) accounted for 94.76–98.50% of the total 22 microelements in the turnips.

In addition, the harmful trace elements accumulated in the root of turnip were lower than those of the leaf and stem, and also lower than the threshold values compared with the limit values of the “General Standard for Contaminants and Toxins in Food and feed—CODEX STAN 193–1995 (FAO/WHO)” (Pb 0.3 mg/kg FW for Brassica vegetables, Cd 0.1 mg/kg FW for the root and tuber vegetables, and 0.05 for Brassica vegetables), combined with “National Food Safety Standard Maximum Levels of Contaminants in Foods” of China (GB 2762–2017, Cr 0.5 mg/kg FW and As 0.5 mg/kg FW). Otherwise, the content of Cd in the stem (0.19 to 0.48 mg/kg DW) and leaf (0.23 to 0.85 mg/kg DW) as well as the Cr and Pb contents in the leaf (0.96 to 2.67 mg/kg DW, 0.57 to 1.96 mg/kg DW, respectively) were all below the corresponding threshold values.

### 3.2. Soluble Sugar Contents

**Figure 2:**
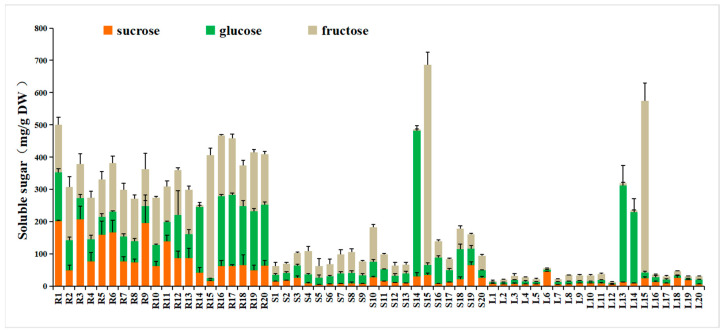
Content of soluble sugar constituents in 3 plant parts of 20 turnip strains. (R: root; S: stem; L: leaf).

The total soluble sugar (TSS) content in turnip ranged from 12.86 to 686.84 mg/g DW of different parts, with an order of root > stem > leaf in general except for strains 13, 14, and 15 (Figure 2). Three soluble sugars, including a disaccharide (sucrose) and two monosaccharides (glucose, fructose), were measured. The order of these three sugar components varied in different strains. Notably, the content of fructose exhibited a very high value in strain 15, and the content of glucose exhibited a very high value in the stem and leaf of strain 14, as well as in the leaf of strain 13. Additionally, the content of sucrose exhibited a relatively high value in the root of strains 1, 3, and 9, which has the highest sweetness among the three sugar components.

Moreover, the average contents of sucrose were 97.10 mg/g DW, 17.96 mg/g DW, and 11.94 mg/g DW in the root, stem, and leaf, respectively; the average contents of glucose were 111.17 mg/g DW, 57.32 mg/g DW, and 33.78 mg/g DW in the root, stem, and leaf, respectively; and the average contents of fructose were 147.98 mg/g DW, 74.44 mg/g DW, and 36.61 mg/g DW in root, stem, and leaf, respectively.

### 3.3. Free Amino Acids Contents

Seventeen free amino acids (FAAs) were measured in this work, namely Asp, Ser, Glu, Gly, His, Arg, Thr, Ala, Pro, Cys, Tyr, Val, Met, Lys, Ile, Leu, and Phe (Table S2), and FAAs are classified as indispensable amino acids (such as Thr, Phe, Lys, Leu, Met, Val, and Ile) and dispensable amino acids.

**Figure 3:**
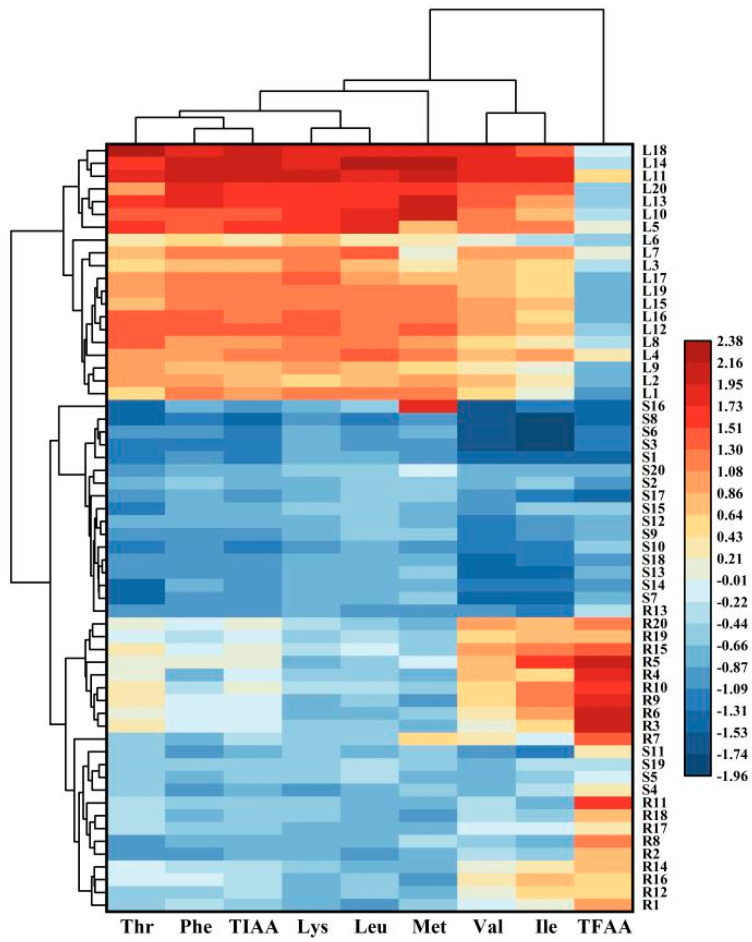
Cluster heat map of TIAA, TFAA, and 7 indispensable AA components in 3 plant parts of different turnip strains. “TIAA” means total indispensable amino acid; “TFAA” means total free amino acid. (R: root; S: stem; L: leaf).

The total content of the 17 FAAs ranged from 16.17 to 56.85 mg/g DW, while the content of 7 indispensable AAs ranged from 1.57 to 8.24 mg/g DW and accounted for 6.05–28.08% of total FAAs. The profiles of amino acids of the turnip plant showed obvious differences among the three parts (Figure 3). The total FAA content in the root was higher than those in the leaf and stem, and the content exhibited a very high value in the root of strains 3 and 5. Interestingly, the content of total indispensable AAs in the leaf was higher than that in the root, followed by the stem. In the leaves of strains 11 and 14, the content of the total indispensable AAs exhibited a relatively high value.

### 3.4. Total Polyphenol and Total Flavonoid Contents

The range of TF content was from 2.92 to 68.49 mg/g DW, with an average value of 4.83 mg/g DW in the root, 11.45 mg/g DW in the stem, and 57.32 mg/g DW in the leaf (Table 1). Meanwhile, the content of TP in turnip was from 1.48 to 12.47 and the average value of the leaf was 11.66 mg/g DW, which was higher than those in the root and stem. There is an obvious tight relationship between TP and TF contents in the stem (R^2^ = 0.62, Figure S1); however, there is only a small correlation with the root and leaf.

In addition, the content of TP in the leaf of strains 4, 8, 13, 16, and 17 was excellent, with a value of over 60 mg/g DW, and the highest value of TP content in the root and stem was from strains 10, 11, 12, 13, and 16 and strains 2, 9, 15, 16, and 17, respectively. At the same time, the TF content in the leaf of strains 2, 9, 12, 14, and 15 was above 12 mg/g DW, and the highest values in the root and stem were from strains 5, 6, 12, 15, and 16 and strains 2, 12, 16, 17, and 19, respectively.

### 3.5. Glucosinolate Profiles

**Figure 4:**
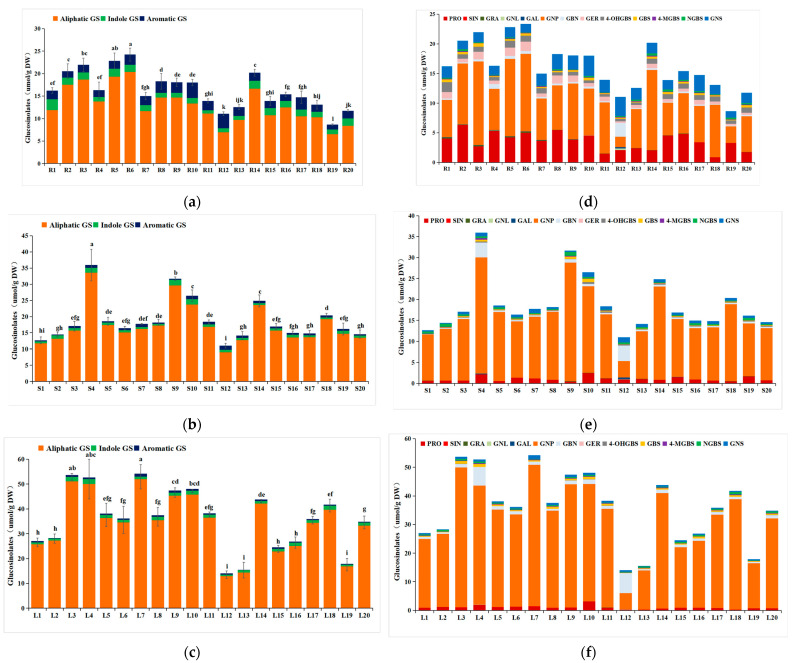
The content of total aliphatic, indole, and aromatic GS ((**a**): root; (**b**): stem; (**c**): leaf), and individual GSs ((**d**): root; (**e**): stem; (**f**): leaf) in 3 plant parts of 20 turnip strains. (R: root; S: stem; L: leaf). Data of the total GS content were analyzed using one-way ANOVA followed by Duncan’s test in Figure 1a–c and different letters indicate significant differences in content among different strains (*p* < 0.05).

The range of the total GS (TG) content of the 20 strains was 8.66–54.19 μmol/g DW, with the highest value in the leaf of strain 7 (Figure 4c) with no significant difference between strain 3 and strain 4, and the lowest value in the root of strain 19 (Figure 4a). Notably, the TG content differed markedly among the different parts of the turnip plant. The TG content in the leaf ranged from 14.09 to 54.19 umol/g DW, and the ranges of the stem and root were from 11.00 to 35.95 umol/g DW and 8.66 to 24.24 umol/g DW, respectively (Figure 4a–c), with an average value of 16.33 μmol/g DW, 18.77 μmol/g DW, and 35.82 μmol/g DW in the root, stem, and leaf, respectively.

Thirteen individual GSs (IGs) were detected in twenty turnip strains, which could grouped into three classes: (1) Aliphatic GS, including progoitrin (PRO), sinigrin (SIN), gluconapoleiferin (GNL), glucoalyssin (GAL), gluconapin (GNP), glucoerucin (GER), glucobrassicanapin (GBN), and glucoraphanin (GRA); (2) Indole GS, including 4-hydroxyglucobrassicin (4-OHGBS), glucobrassicin (GBS), 4-methoxyglucobrassicin (4-MGBS), and neoglucobrassicin (NGBS); and (3) Aromatic GS, including gluconasturtiin (GNS), respectively (Figure 4d–f).

In the leaf, the content of total aliphatic GSs was accounted for in 92.17–96.39% of the TG, while total indole GSs and total aromatic GSs were accounted for in 1.72–5.96% and 0.64–4.15%, respectively. The values in the root and stem were 62.70–85.26% (aliphatic GS in the root), 5.29–14.83% (indole GS in the root), and 7.03–29.05% (aromatic GS in the root), and 82.10–95.03% (aliphatic GS in the stem), 2.55–7.27% (indole GS in the stem), and 1.08–11.34% (aromatic GS in the stem), respectively.

The most abundant individual GS in the 20 turnip strains was GNP, accounting for 31.94–67.40%, 35.63–90.01%, and 40.00–92.51% of the TG in the root, stem, and leaf, respectively, followed by PRO and GNS (root and stem)/GBN (leaf). In addition, the contents of SIN, GNL, and GAL were at a very low level in most of the strains.

### 3.6. Assessment Using PCA, FA, and HCA

**Figure 5:**
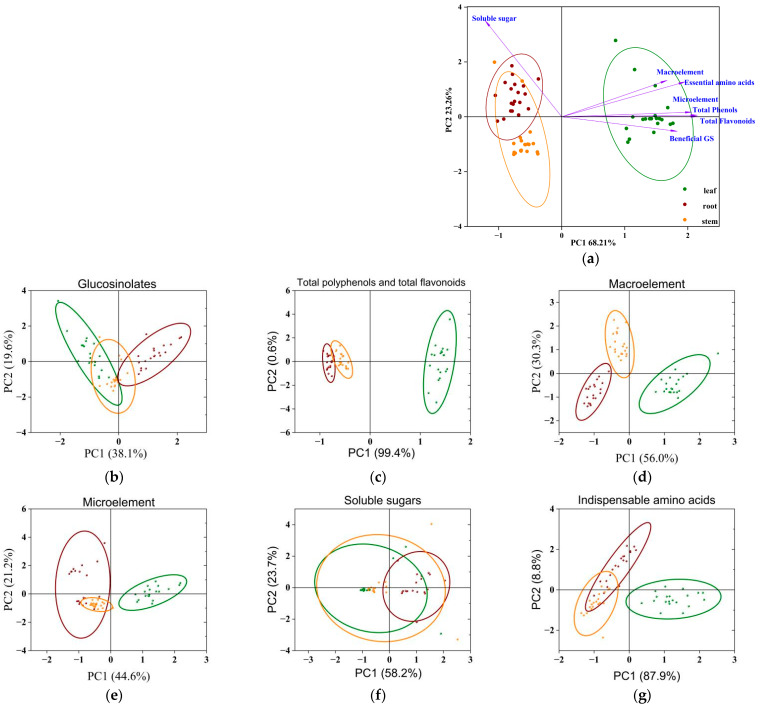
Plots of PCA scores for compounds analyzed in plant parts of turnip strains. (**a**) All compounds; (**b**) glucosinolates (except PRO); (**c**) total polyphenols and total flavonoids; (**d**) macroelement; (**e**) microelement; (**f**) soluble sugars; (**g**) indispensable amino acids.

The relationships between the variable compounds, including beneficial GSs (except PRO), total flavonoids, total phenols, macroelements, microelements, soluble sugars, and indispensable amino acids, were analyzed using PCA for each sample (Figure 5). The results showed that principal component 1 (PC1, 68.21%) and PC2 (23.26%) were responsible for 91.47% of the accumulated variance. Furthermore, PCA was carried out for all profiles contained in each index. The results suggested that the correlations of glucosinolates and soluble sugars among three different parts were stronger than the other four components, and the other nutritional indexes showed stronger correlations between the root and stem rather than the root and leaf or stem and leaf of turnip.

Furthermore, the total score was weighted by the variance contribution of components involved in PCA, and the calculation formula was as follows:

Score = 68.21 ÷ 91.47 × FAC1-1 + 23.26 ÷ 91.47 × FAC2-1

(where FAC1-1 and FAC2-1 are the scores of the principal components).

The scores calculated by the FA of different parts of 20 turnip strains are shown in Table S3. Most of the strains exhibited the best nutrition in the leaf. The leaf of strains 4, 7, and 11; the stem of strains 4, 9, and 2; and the root of strains 3, 10, and 5 gained the highest scores in the leaf, stem, and root, respectively.

Strain 7 (male parent) and strains 14, 11, 19, 20, and 15 (female parents) were crossed to obtain offsprings 1, 13, 16, 12, and 17, respectively. All metabolites of eleven turnip strains containing kinship in three parts were analyzed by hierarchical clustering analysis (HCA) (Figure S2), and the relative distance between parents and offspring was further calculated. The results showed that R1, S1, R13, S12, R17, S17, and L17 were closer to the male parent, while R16, S16, L16, L1, L13, R12, and L12 were closer to the female parent, and S13 had a similar distance to both.

## 4. Discussion

Turnip was a source of diverse GSs, especially aliphatic GSs, and the total GS content among plant parts and strains was varied, which is consistent with Dejanovis et al. [24]. The total GS content of different plant parts in this work is similar to the previous studies on turnip leaves (19.50–36.34 μmol/g DW) [25] and roots (12.6–26.0 μmol/g DW) [26], and higher than those in cabbage leaves (3.99–23.75 μmol/g DW) [27] and kale leaves (0.66–8.03 μmol/g DW) [28]. Mostly, the TG content in the leaf was highest, while it varied for the stem and root among different strains. For example, the content of TG in the root was higher than those in the stem in strains 1, 2, 3, 5, 6, 8, 12, and 16, while in the other strains, the opposite observation was made. This is also different from the results of pak choi [29], in which the results showed that the TG content in the root was higher. The differences between species and organs may be owed to the genetic impact of selective breeding on edible parts and consumption patterns. The high content of GSs is always related to the resistance to diseases and insect pests, and it also produces an excessively bitter or pungent taste in part [30].

Three classes including thirteen individual GSs were detected in this work. In general, the proportion of aliphatic GSs was evidently higher than those of indole GSs and aromatic GSs. Similarly to most of the Brassica species, the total aliphatic GSs were exhibited in the predominant groups in turnip, which is consistent with the results of Cartea et al. [23]. There are more IG types in turnip compared to other Brassica species, such as Chinese cabbage (8 IGs) and pak choi (12 IGs) [31], and the GNP, PRO, and GNS/GBN have a relatively high content in different parts. In order to achieve cultivars with different metabolite profiles, it is crucial to have a diversity of GSs in the vegetable material of the turnip (e.g., leaves).

Previous studies indicated that GNS exhibits anti-microbial and anti-nematode effects [32], while GBS, GRA, and GER demonstrate various biological activities that are indirectly linked with cancer prevention [30]. Meanwhile, GNP, GBN, and GNS are commonly associated with a unique taste in vegetables [30]. In contrast, PRO is thought to be toxic to animals and could cause goiter [33]. In the root, the PRO content of strains 18, 11, 20, 14, and 12 was lowest; however, it was still higher than that of all of the samples from the leaf and stem. Otherwise, the sum of the other 12 individual GS contents was compared and the results showed that (1) in the leaf, strains 7, 3, 4, 9, 10, 14, and 18 had higher values, which were more than 40 μmol/g DW; (2) in the root, strains 6, 3, 5, 14, and 9 had the higher values of above 14.22 μmol/g DW; and (3) in the stem, strains 4, 9, 10, 14, and 18 had the higher values (19.87–33.80 μmol/g DW).

The contents of flavonoids and other polyphenol substances have been suggested to play a preventive role in the development of cancer, cardiovascular disease, and other chronic diseases [34]. The results showed that the TF content in the stem and leaf was higher than that of *Brassica juncea* (9.4 mg/g DW) [35]. The content of TP in turnip was lower than that of TF, which might be due to the different extract reagents used [36], and the average value of the leaf was similar to that of broccoli (12.85 mg/g DW) and higher than that of cauliflower (5.83 mg/g DW) [37]. There was a significant positive correlation between TP and TF contents in turnip stem, which is consistent with the results of Heimler et al. [37], who showed a good correlation (R^2^ = 0.974) between the TP and TF contents in broccoli and Italian kale. Furthermore, the TP and TF contents in the leaf were obviously higher than those in the stem and root. This agrees with the result reported by Bouslimi et al. [35] that the TP and TF content of aerial parts in *Brassica juncea* or *Cakile maritima* are higher than those of the root.

Macroelements are of great importance in human health, which are required by an adult in large amounts [38]. The total content of six macroelements in turnip was similar to that in cabbage (20.34–185.88 g/kg DW) [39]. K and Na are essential minerals for the maintenance of cellular homeostasis, the protection of muscles and nerve health, and the regulation of cell osmotic pressure [38], which were at a higher level in the root of strain 12. P is vital for the metabolism of proteins, and plays a critical function as part of the hydroxyapatite in the skeleton and as a substrate for ATP synthesis [40], which was at a higher level in the root of strains 20, 15, and 12. Ca is required for constituting healthy bones and teeth, and many vital physiological functions including signaling, enzyme regulation, and muscle contraction [38], which was at a higher level in the root of strains 20, 10, and 4. Mg plays an important role in the physiological functions of the cardiovascular and neurological systems and muscles [38], which was at a higher level in the root of strains 20, 18, and 11. S is an important component of proteins in the human body, related to human pathologies/malignancies [41], which was at a higher level in the root of strains 10, 18, and 12.

For energy supply, microelements cannot be substituted, as they cannot be synthesized in the human body. Consequently, microelement homeostasis is closely regulated by diet [38]. It seems that turnip is rich in Fe, Zn, Al, and Mn, and this result is consistent with kale [42]. Interestingly, macroelements are in higher abundance in the stem, while there are more microelements in the leaf. Notably, Fe was the most abundant micronutrient detected in turnip, which plays an important role in the transportation of oxygen, several oxidation–reduction pathways, and cellular growth [23], and its average content was 47.51 mg/kg DW, 34.93 mg/kg DW, and 151.72 mg/kg DW in the root, stem, and leaf, respectively. The content exhibited a higher value in the leaf of strains 1, 7, and 13. Zn is an integral constituent of insulin and is essential for thymic functions [23]. The average content of Zn was 44.16 mg/kg DW, 62.23 mg/kg DW, and 96.20 mg/kg DW in the root, stem, and leaf, respectively, and the content exhibited a higher value in the leaf of strains 2, 11, and 13, as well as in the stem of strain 2. Mn is associated with bone development and is involved in amino acid, lipid, and carbohydrate metabolism [23]. The average content of Mn was 8.05 mg/kg DW, 16.39 mg/kg DW, and 66.11 mg/kg DW in the root, stem, and leaf, respectively, and the content exhibited a higher value in the leaf of strains 1, 7, and 13. Excessive Al can lead to memory loss and osteoporosis, and can affect development [43]. The average content of Al was 33.03 mg/kg DW, 20.94 mg/kg DW, and 119.20 mg/kg DW in the root, stem, and leaf, respectively. This is lower than that of cabbage (385.6 mg/kg DW) [39], and is the highest value exhibited in the leaf of strains 15, 12, and 2.

In general, the consumption of those strains is normally safe due to the level of harmful trace elements accumulated in the root of turnip being lower than the limit values of the “General Standard for Contaminants and Toxins in Food and feed—CODEX STAN 193–1995 (FAO/WHO)” and “National Food Safety Standard Maximum Levels of Contaminants in Foods (China, GB 2762–2017)”. The root is the main edible part of turnip.

Plant sugars are primary metabolites that provide energy storage and play a crucial role in signaling pathways in plant growth and development [44]. On the whole, the average content of the TSS of turnip was 356.25 mg/g DW, 149.73 mg/g DW, and 82.33 mg/g DW in the root, stem, and leaf, respectively, which is higher than that of Indian mustard (around 60 mg/g DW) [45] and similar to those of cauliflower (192.0–224.4 mg/g DW) and broccoli (214.3–241.6 mg/g DW) [46]. Schonhof et al. [47] indicated that the high content of TSS could modify the contents of bitter glucosinolates in Brassica crops, which could promote human consumption. The TSS content exhibited a very high value in the stem and leaf of strains 14 and 15, as well as in the root of strain 13.

The sweetness of vegetables and fruit depends not only on the total amount of sugar but also on the sugar composition [48]. The average content of TSS and each component were highest in the turnip root, followed by the stem, and finally, the leaf, except for strains 13, 14, and 15. When compared with the swede roots (*Brassica napus* L.), the contents of sucrose (2.4 g/100 g DM) was lower than in our turnip roots, while the contents of glucose (30.6 g/100 g DM) and fructose (20.9 g/100 g DM) were higher [49].

Free amino acids, as well as peptides, make an important contribution to taste [50], and aromatic amino acids, Cys, and Met are often precursors of odorants for the formation of aroma compounds and branched chain volatiles in plants according to Wüst [51]. In spinach, Yoon et al. [52] recorded a lower total FAA content (10.28–20.82 mg/g DW), and a similar proportion of total indispensable AAs (9.70–18.28%) compared to our research. Otherwise, Tang et al. [53] showed that an appropriate dietary intake of Thr can promote animal growth, enhance immune function, and maintain intestinal health. Bingol et al. [54] found that Asp, Val, and Pro affect color density and anthocyanin stability. In this work, Thr and Val were the highest constituents among seven indispensable AAs, which accounted for 17.77–29.56% and 15.46–31.27%, respectively. We found that Val had the highest proportion (19.5–27.2% of the total indispensable AAs) in spinach [52]. The content of Thr exhibited a relatively high value in the leaf of strains 11 and 18, as well as in the leaf of strains 11 and 14. In addition, Rose [55] found that sulfur-containing amino acids (Met and Cys) benefited age-related diseases. Meanwhile, Met is present in all parts of turnips, which accounted for 0.83%–9.99% of indispensable AAs, while Cys is widely present in the root with a content of up to 13.82% of indispensable AAs, which are not detected on the leaf and stem in some strains.

According to PCA, the absolute values of the coefficients of macroelements, total flavonoids, total phenols, microelements, beneficial GS, and indispensable AAs in PC1 were more than 0.5, and greater than another index. Hence, PC1 was a comprehensive reflection of the six indexes, indicating that these were indispensable in evaluating the nutritional quality value of turnip. In relation to PC2, the soluble sugar is the variable with the highest positive weight. A closer relationship was shown between the stem and root, especially for TP and TF content and mineral elements. This phenomenon might be applied to variety screening in breeding; however, further experimental verification is still needed.

Otherwise, the scores of different parts from the same turnip strain, which were weighted by the variance contribution of each component, varied greatly. The score of the turnip leaf was higher than those of the stem and root, due to higher microelements, TF, and TP, indispensable AAs, and higher GS contents. Strains 4, 7, 11, 14, and 10 were excellent due to their highest top five scores, while strains 4 and 10 seemed better if considering the performance in all three different parts. It is suggested that different strains might be used for different purposes. In addition, the relative distance between parents and offspring according to HCA indicated that the intricate metabolic mechanisms or these pairwise strains likely had very similar genetic backgrounds, resulting in the metabolites of turnip having no clear associations between parent–child generations.

## 5. Conclusions

In this work, a comprehensive list of bioactive compounds with potential beneficial effects to human health was systematically analyzed in 20 turnip strains of three parts. The profiles of nutrient compounds in different parts of the 20 strains varied, and most of the turnip strains had better nutrition in the leaf, followed by the stem and root. There was a stronger correlation between the root and stem compared to the root and leaf or the stem and leaf. Hence, more studies are required to clarify the mechanism responsible for different plant organs transporting metabolites, and how these metabolites are transformed under biological or abiotic stress. Furthermore, there was a certain correlation between the parents and offspring of metabolites, although there is not always a convincing relationship. There are internal and external factors that influence the biosynthesis and degradation of metabolites, and the mechanisms still need further studies, including but not limited to genetic and environmental response.

The findings of this study provide the basis for using the different parts of the turnip in accordance with the composition, especially turnip leaf use in human food and animal feeding due to higher contents of microelements, total indispensable amino acids, and total GS with lower PRO proportion, and in medicinal purposes due to higher TP and TF, as well as a higher GS content. Meanwhile, the root seems to be a good source of soluble sugar and total free amino acid content in most strains. On the other hand, strains 4 and 10, as well as 7, 11, and 14, gained higher scores in FA, which suggested that these strains may be worth subsequent breeding research or product production.

## Figures and Tables

**Table 1:** Contents of total flavonoids (TF) and total phenols (TP) in 3 plant parts (R: root, S: steam, L: leaf) of 20 strains. Data are expressed as mean ± standard deviation (n = 3).

Strain	TF (mg Rutin/g DW)			TP (mg Gallic Acid/g DW)		
	R	S	L	R	S	L
1	2.92 ± 0.34 ^k^	8.44 ± 0.89 ^i^	47.30 ± 3.20 ^f^	2.29 ± 0.16 ^bcde^	2.55 ± 0.13 ^h^	11.55 ± 0.31 ^abcde^
2	3.10 ± 0.47 ^jk^	12.47 ± 0.40 ^abc^	49.55 ± 4.87 ^ef^	2.27 ± 0.34 ^bcde^	3.81 ± 0.76 ^abcd^	12.47 ± 0.93 ^a^
3	3.92 ± 0.34 ^ij^	10.34 ± 2.27 ^defghi^	52.09 ± 7.57 ^def^	2.35 ± 0.14 ^bcd^	2.56 ± 0.58 ^gh^	10.91 ± 0.29 ^cde^
4	4.07 ± 0.45 ^hi^	10.86 ± 1.31 ^cdefgh^	62.72 ± 3.51 ^ab^	1.48 ± 0.16 ^f^	3.12 ± 0.36 ^defgh^	11.96 ± 0.41 ^abcd^
5	4.26 ± 0.47 ^ghi^	10.01 ± 0.89 ^ghi^	55.46 ± 1.91 ^cde^	2.88 ± 0.18 ^a^	2.55 ± 0.13 ^hc^	11.73 ± 0.74 ^abcde^
6	3.07 ± 0.49 ^jk^	10.19 ± 0.61 ^efghi^	55.82 ± 1.75 ^cde^	2.54 ± 0.27 ^ab^	3.30 ± 0.25 ^bcdef^	11.02 ± 0.36 ^bcde^
7	4.07 ± 0.22 ^hi^	12.43 ± 0.71 ^abcd^	57.77 ± 1.58 ^bcd^	2.10 ± 0.46 ^bcde^	3.25 ± 0.21 ^bcdefg^	10.84 ± 0.91 ^de^
8	5.60 ± 0.39 ^bcde^	11.87 ± 1.73 ^bcdefg^	60.24 ± 5.03 ^bc^	2.00 ± 0.37 ^cde^	3.04 ± 0.40 ^efgh^	11.82 ± 0.25 ^abcde^
9	4.56 ± 0.39 ^fghi^	12.72 ± 1.30 ^abc^	59.71 ± 2.31 ^bc^	2.42 ± 0.26 ^bcd^	3.31 ± 0.21 ^bcdef^	12.47 ± 0.53 ^a^
10	6.57 ± 0.57 ^a^	10.75 ± 0.40 ^cdefgh^	55.99 ± 0.25 ^bcde^	2.47 ± 0.22 ^abc^	3.31 ± 0.27 ^bcdef^	11.98 ± 0.38 ^abg^
11	6.13 ± 0.50 ^abcd^	9.18 ± 0.34 ^hi^	59.73 ± 3.48 ^bc^	2.44 ± 0.13 ^abcd^	3.18 ± 0.18 ^cdefgh^	11.51 ± 0.35 ^abcde^
12	6.31 ± 0.33 ^abc^	12.24 ± 0.81 ^abcde^	58.72 ± 0.77 ^cde^	2.50 ± 0.38 ^ab^	3.91 ± 0.33 ^ab^	12.42 ± 0.58 ^a^
13	6.46 ± 0.36 ^ab^	12.13 ± 1.47 ^abcdef^	60.14 ± 1.15 ^bc^	2.15 ± 0.10 ^bcde^	3.10 ± 0.56 ^defgh^	11.79 ± 0.36 ^abcde^
14	5.12 ± 0.91 ^efg^	11.04 ± 0.55 ^bcdefgh^	54.77 ± 7.19 ^cde^	2.48 ± 0.15 ^abs^	3.49 ± 0.22 ^abcde^	12.09 ± 0.41 ^ab^
15	4.89 ± 0.23 ^efgh^	12.69 ± 1.07 ^abc^	53.97 ± 0.80 ^cde^	2.50 ± 0.05 ^ab^	2.74 ± 0.18 ^fgh^	12.04 ± 0.72 ^abg^
16	6.28 ± 0.28 ^abc^	14.04 ± 0.51 ^a^	68.49 ± 0.43 ^a^	2.49 ± 0.10 ^ab^	3.91 ± 0.48 ^ab^	11.34 ± 0.66 a^bcde^
17	5.45 ± 0.34 ^cdef^	13.06 ± 0.62 ^ab^	60.68 ± 2.20 ^bc^	1.88 ± 0.21 ^e^	4.06 ± 0.19 ^a^	11.54 ± 0.67 ^abcde^
18	5.53 ± 0.96 ^bcde^	10.08 ± 1.71 ^fghi^	54.57 ± 4.04 ^cde^	2.03 ± 0.18 ^cde^	3.17 ± 0.34 ^cdefgh^	10.76 ± 0.38 ^e^
19	3.07 ± 0.40 ^jk^	12.21 ± 0.87 ^abcde^	59.70 ± 2.84 ^bc^	2.30 ± 0.04 ^bcde^	3.87 ± 0.48 ^abc^	11.56 ± 0.94 ^abcde^
20	5.23 ± 0.85 ^def^	12.28 ± 0.85 ^abcde^	58.96 ± 2.56 ^bc^	1.99 ± 0.27 ^de^	3.26 ± 0.19 ^bcdefg^	11.48 ± 0.49 ^abcde^

Note: Data were analyzed by one-way ANOVA followed by Duncan’s test. Different letters indicate significant differences in content among different strains (*p* < 0.05).

## Data Availability

The data presented in this study are available.

## Supplementary Materials

The following supporting information can be downloaded at https://www.mdpi.com/article/10.3390/foods12173195/s1, Figure S1: Total phenol and total flavonoid correlation analysis in three plant parts of 20 turnip strains; Figure S2: Hierarchical clustering analysis (HCA) on 11 turnip strains containing kinship in three parts; Table S1: The details of 20 different turnip strains; Table S2: Free amino acid composition and content in three plant parts of 20 turnip strains; Table S3: Integrate score of 20 turnip strains.
