# Supplementary material for: Chemical Profile of Turnip According to the Plant Part and the Cultivar: A Multivariate Approach
Source: Foods. 2023 Aug 24;12(17):3195. doi: 10.3390/foods12173195 (PMC10486609; doi:10.3390/foods12173195)
Supplement: Supplementary file 1 [file foods-12-03195-s001.zip › foods-2520125-supplementary.pdf]

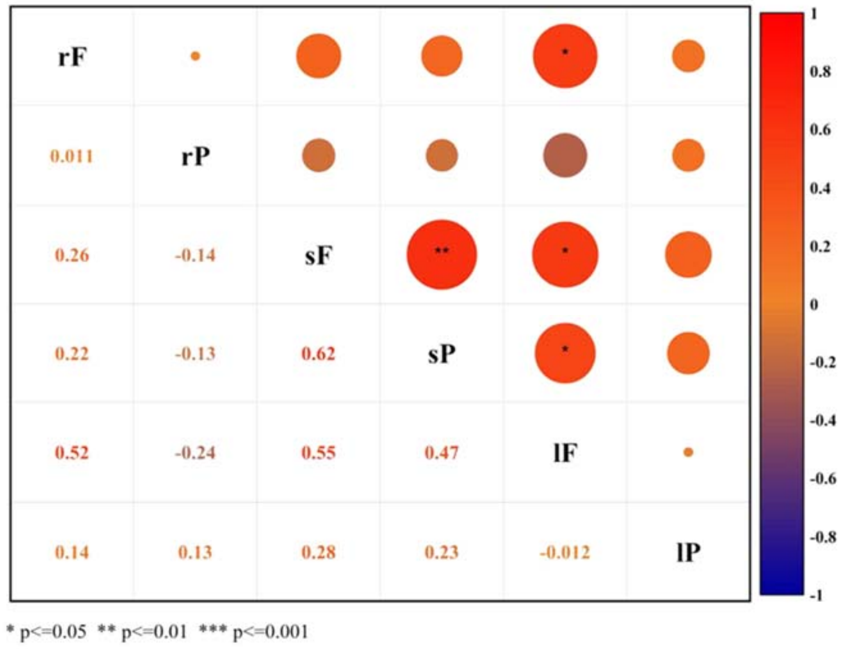

Figure S1. Total phenols and total flavonoids correlation analysis in three plant parts of 20 turnip strains.

Note: “rF” means total flavonoids in root; “rP” means total phenols in root; “sF” means total flavonoids in stem; “sP” means total phenols in stem; “lF” means total flavonoids in leaf “lP” means total phenols in leaf.

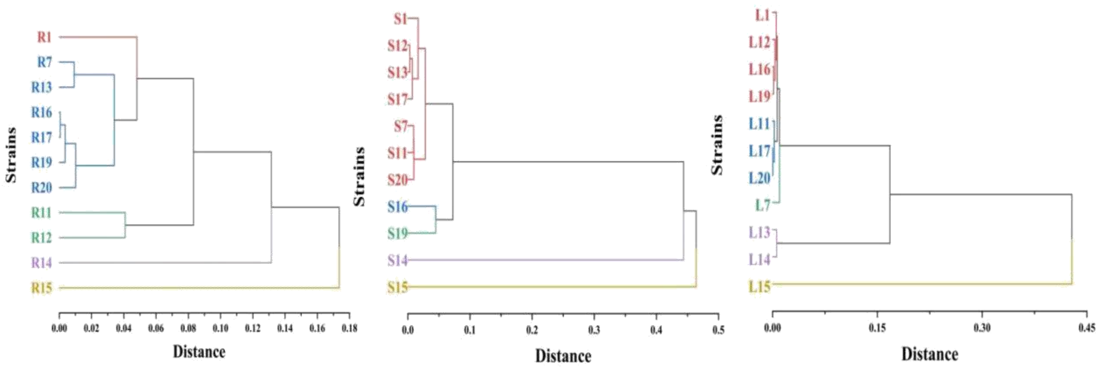

Figure S2. Hierarchical clustering analysis (HCA) on 11 turnip strains containing kinship in three parts.

Note: Strain 7 (female parent) and strain 14, 11, 19, 20, 15 (male parents) were crossed to obtain offspring 1, 13, 16, 12, 17, respectively.

Table S1. The details of 20 different turnip strains

No.	Strain
1	wencheng×2F
2	17P-48
3	SQ×taishunhong
4	17P-16
5	16p-390×2F
6	16p-380×2F
7	2F
8	16p-313×2F
9	16p-373×2F
10	17p-72
11	taishunhong
12	nanxi ×2F
13	taishunhong×2F
14	wencheng
15	wenzhou
16	yuhuan×2F
17	wenzhou×2F
18	16p-313×taishunhong
19	yuhuan
20	nanxi

Table S2. Free amino acid composition and content in three plant parts of 20 turnip strains (mg/g DW)

Sample	Asp	Ser	Glu	Gly	His	Arg	Thr	Ala	Pro	Cys	Tyr	Val	Met	Lys	Ile	Leu	Phe	TEAA	TFAA
R1	1.75±0.51	3.97±0.38	1.16±0.63	0.39±0.06	25.80±2.49	0.36±0.07	0.74±0.13	3.33±0.68	3.56±1.83	0.21±0.05	0.36±0.03	0.92±0.06	0.05±0.03	0.26±0.02	0.59±0.07	0.24±0.01	0.42±0.08	3.21±0.24	44.09±3.98
R2	1.85±0.52	3.32±1.12	1.55±0.61	0.34±0.19	25.05±11.44	0.38±0.11	0.60±0.08	2.48±2.69	3.52±1.37	0.20±0.12	0.30±0.05	0.82±0.20	0.04±0.01	0.26±0.02	0.46±0.16	0.22±0.00	0.21±0.19	2.61±0.61	41.61±14.86
R3	1.29±0.04	4.37±0.21	2.72±0.05	0.51±0.02	35.40±2.56	0.40±0.04	1.05±0.08	2.99±0.37	3.44±0.33	0.23±0.03	0.44±0.02	1.00±0.09	0.04±0.01	0.41±0.02	0.67±0.04	0.32±0.02	0.43±0.02	3.93±0.28	55.73±2.39
R4	2.94±1.06	4.98±0.65	2.77±0.59	0.32±0.21	33.39±4.82	0.52±0.10	0.97±0.13	1.53±0.50	1.93±0.77	0.35±0.05	0.41±0.03	1.19±0.12	0.04±0.01	0.44±0.06	0.66±0.09	0.35±0.04	0.29±0.03	3.94±0.44	53.09±6.12
R5	1.86±0.39	4.58±0.38	1.70±0.28	0.66±0.02	32.99±2.37	0.37±0.02	1.00±0.09	4.57±0.61	4.97±0.57	0.33±0.02	0.48±0.09	1.20±0.13	0.08±0.04	0.31±0.03	0.84±0.14	0.36±0.02	0.52±0.15	4.33±0.53	56.85±1.23
R6	1.80±0.07	4.52±0.47	2.13±0.21	0.68±0.02	30.52±2.77	0.44±0.14	1.02±0.07	3.77±0.54	6.59±0.86	0.51±0.09	0.43±0.05	1.03±0.06	0.06±0.02	0.29±0.03	0.72±0.05	0.29±0.04	0.44±0.07	3.86±0.33	55.25±2.02
R7	1.59±0.11	4.63±0.43	1.98±0.10	0.41±0.02	30.50±2.43	0.59±0.06	0.75±0.04	2.57±0.82	1.13±0.56	0.34±0.05	0.36±0.03	1.01±0.10	0.13±0.01	0.43±0.02	0.56±0.07	0.34±0.02	0.26±0.01	3.49±0.14	47.59±4.25
R8	3.89±0.25	3.36±0.68	2.67±0.22	0.44±0.03	27.39±2.18	0.27±0.06	0.60±0.07	2.31±0.95	1.57±1.56	0.38±0.03	0.28±0.04	0.75±0.09	0.07±0.01	0.33±0.03	0.44±0.05	0.28±0.02	0.28±0.03	2.74±0.14	45.30±3.54
R9	1.77±0.09	4.53±0.15	2.55±0.49	0.62±0.05	31.49±3.05	0.39±0.08	1.09±0.13	3.89±0.54	3.99±1.46	0.48±0.02	0.45±0.07	1.12±0.11	0.03±0.01	0.32±0.02	0.76±0.07	0.32±0.02	0.45±0.06	4.09±0.37	54.23±5.03
R10	3.07±0.05	3.21±0.30	4.58±0.05	0.41±0.04	27.54±3.34	1.06±0.04	1.07±0.07	3.15±0.33	3.84±1.24	0.23±0.01	0.46±0.07	1.11±0.04	0.05±0.00	0.47±0.05	0.77±0.06	0.38±0.03	0.42±0.06	4.27±0.22	51.82±4.33
R11	3.07±1.14	4.48±0.24	9.61±0.10	0.26±0.02	23.78±1.08	0.80±0.10	0.86±0.13	1.85±0.11	3.09±0.33	0.11±0.02	0.35±0.03	0.81±0.05	0.04±0.01	0.41±0.03	0.42±0.03	0.26±0.00	0.30±0.05	3.11±0.13	50.52±0.56
R12	1.55±0.43	3.67±0.98	2.65±1.29	0.30±0.08	18.90±5.07	0.60±0.18	0.73±0.33	3.20±0.44	3.63±1.48	0.20±0.05	0.41±0.14	0.96±0.27	0.04±0.02	0.36±0.05	0.64±0.20	0.33±0.09	0.36±0.15	3.42±1.10	38.54±9.13
R13	1.49±0.49	2.81±1.27	4.68±1.70	0.17±0.06	13.36±6.52	0.41±0.22	0.59±0.37	1.31±0.64	2.08±0.74	0.07±0.03	0.26±0.12	0.60±0.25	0.03±0.02	0.29±0.09	0.36±0.14	0.22±0.07	0.22±0.11	2.30±1.03	28.95±12.68
R14	2.46±0.11	3.75±0.29	1.00±0.13	0.45±0.04	23.95±2.16	0.81±0.07	0.94±0.02	2.42±0.42	2.51±0.09	0.33±0.11	0.40±0.01	1.00±0.01	0.04±0.00	0.41±0.04	0.60±0.04	0.30±0.02	0.37±0.01	3.65±0.06	41.72±2.75

R15	3.60±0.94	5.49±0.25	5.07±0.84	0.37±0.06	22.80±1.04	0.76±0.07	1.08±0.07	3.38±0.39	2.73±0.26	0.10±0.06	0.50±0.07	1.24±0.13	0.05±0.01	0.49±0.04	0.78±0.09	0.47±0.06	0.48±0.06	4.59±0.46	49.39±3.06
R16	1.84±0.47	3.89±0.39	2.90±0.59	0.28±0.03	18.55±1.07	0.64±0.10	0.90±0.05	1.84±0.61	3.15±1.16	0.38±0.02	0.48±0.09	1.01±0.14	0.03±0.00	0.34±0.13	0.68±0.08	0.34±0.05	0.46±0.10	3.77±0.36	37.71±1.95
R17	2.50±0.16	3.70±1.09	2.70±1.37	0.26±0.03	19.70±6.03	0.41±0.14	0.79±0.27	1.92±0.71	1.17±0.33	0.19±0.04	0.36±0.07	0.89±0.16	0.04±0.02	0.34±0.03	0.53±0.12	0.28±0.05	0.31±0.05	3.20±0.68	36.09±10.06
R18	2.31±0.47	3.10±0.59	6.63±1.15	0.21±0.10	21.66±6.08	0.63±0.11	0.79±0.17	1.72±0.57	1.56±0.70	0.14±0.04	0.36±0.07	0.79±0.17	0.03±0.01	0.40±0.09	0.48±0.11	0.26±0.04	0.28±0.06	3.03±0.48	41.35±8.10
R19	1.97±0.12	4.18±0.86	4.82±2.24	0.40±0.04	17.44±4.32	0.62±0.13	0.87±0.15	3.34±0.64	4.37±2.02	0.19±0.07	0.40±0.02	1.12±0.17	0.06±0.01	0.45±0.10	0.70±0.14	0.40±0.06	0.42±0.01	4.02±0.43	41.75±9.63
R20	3.22±0.93	4.39±0.31	5.71±2.86	0.30±0.12	21.45±2.91	0.92±0.18	1.03±0.14	2.41±0.49	1.42±0.91	0.24±0.06	0.38±0.27	1.29±0.23	0.04±0.01	0.48±0.03	0.70±0.31	0.34±0.11	0.46±0.16	4.34±0.79	44.79±5.39
S1	2.08±0.30	1.85±0.13	3.06±1.28	0.03±0.03	5.06±3.28	0.46±0.08	0.47±0.10	0.62±0.38	0.92±0.28	0.02±0.03	0.18±0.01	0.47±0.03	0.03±0.01	0.30±0.04	0.30±0.01	0.27±0.05	0.23±0.05	2.07±0.09	16.34±4.36
S2	3.51±0.25	1.99±0.97	4.16±1.17	0.06±0.04	7.08±6.78	0.60±0.25	0.64±0.19	0.96±0.57	0.61±0.32	0.04±0.06	0.27±0.01	0.66±0.14	0.05±0.02	0.34±0.04	0.46±0.14	0.33±0.02	0.34±0.01	2.82±0.47	22.10±9.98
S3	1.38±0.43	2.00±0.70	3.07±1.01	0.05±0.08	9.05±5.08	0.51±0.06	0.43±0.21	1.16±0.63	0.80±0.39	0.04±0.07	0.14±0.01	0.39±0.07	0.03±0.02	0.29±0.01	0.22±0.03	0.24±0.02	0.16±0.03	1.75±0.23	19.94±8.13
S4	2.13±0.81	3.86±1.00	2.04±0.98	0.17±0.03	20.04±6.70	0.64±0.13	0.70±0.15	1.97±0.35	1.04±0.30	0.10±0.07	0.23±0.06	0.64±0.12	0.05±0.01	0.23±0.20	0.49±0.10	0.30±0.08	0.21±0.07	2.62±0.65	34.84±10.07
S5	3.04±0.57	3.18±1.04	3.67±1.59	0.16±0.05	14.73±7.39	0.88±0.28	0.77±0.09	1.96±0.62	1.06±0.28	0.02±0.03	0.27±0.08	0.68±0.20	0.04±0.01	0.42±0.13	0.48±0.15	0.40±0.13	0.29±0.08	3.08±0.74	32.04±7.74
S6	2.58±0.16	1.76±0.35	5.24±0.35	0.07±0.03	5.24±3.07	0.34±0.11	0.54±0.16	0.82±0.35	1.06±0.21	0.05±0.05	0.16±0.02	0.39±0.04	0.04±0.02	0.27±0.04	0.25±0.06	0.23±0.05	0.18±0.03	1.90±0.24	19.21±4.09
S7	2.11±0.09	2.77±0.05	1.47±0.32	0.14±0.01	11.88±0.24	0.62±0.06	0.42±0.07	1.21±0.07	2.05±0.96	0.17±0.07	0.19±0.05	0.47±0.06	0.05±0.00	0.35±0.05	0.33±0.05	0.29±0.05	0.19±0.05	2.10±0.21	24.70±1.47
S8	1.33±0.64	1.72±0.19	1.89±1.88	0.08±0.02	8.27±2.19	0.41±0.04	0.39±0.19	0.95±0.22	0.48±0.13	0.03±0.05	0.12±0.05	0.34±0.13	0.03±0.01	0.22±0.06	0.24±0.11	0.18±0.05	0.17±0.04	1.57±0.56	16.87±4.67
S9	2.15±0.10	2.78±1.57	2.43±0.67	0.11±0.09	11.02±11.20	0.56±0.33	0.54±0.26	1.40±1.21	0.76±0.10	0.09±0.07	0.20±0.08	0.52±0.17	0.05±0.02	0.32±0.01	0.39±0.22	0.31±0.04	0.23±0.02	2.36±0.68	23.85±15.89
S10	1.79±1	2.16±0	2.07±2	0.10±0	14.55±	0.52±0	0.49±0	1.45±0	0.93±0	0.03±0	0.16±0	0.48±0	0.03±0	0.25±0	0.35±0	0.25±0	0.18±0	2.03±0	25.80±

	.28	.52	.35	.04	3.66	.15	.18	.38	.17	.05	.03	.09	.01	.04	.08	.05	.04	.44	5.33
S11	2.31±0.18	4.62±0.77	4.63±1.66	0.20±0.03	15.80±3.09	0.80±0.15	0.77±0.03	2.31±0.40	1.33±0.33	0.02±0.04	0.19±0.04	0.56±0.09	0.06±0.00	0.37±0.07	0.36±0.09	0.29±0.07	0.20±0.03	2.60±0.36	34.84±2.77
S12	2.23±0.35	2.83±0.82	3.02±1.44	0.11±0.03	10.26±2.68	0.54±0.15	0.65±0.11	1.32±0.49	1.59±0.78	0.03±0.04	0.22±0.04	0.54±0.08	0.04±0.00	0.33±0.05	0.39±0.08	0.32±0.03	0.25±0.03	2.52±0.36	24.68±3.36
S13	2.01±1.31	2.60±0.94	3.12±2.35	0.10±0.06	10.33±5.86	0.53±0.19	0.56±0.23	1.30±0.62	0.90±0.32	0.03±0.05	0.18±0.03	0.47±0.12	0.05±0.02	0.31±0.08	0.33±0.07	0.26±0.07	0.21±0.05	2.18±0.61	23.29±9.75
S14	2.02±0.19	2.46±0.31	0.91±0.23	0.10±0.03	11.84±1.25	0.53±0.04	0.42±0.12	1.13±0.08	0.85±0.30	n.d.	0.20±0.04	0.52±0.09	0.04±0.01	0.34±0.04	0.37±0.04	0.29±0.05	0.25±0.05	2.23±0.37	22.27±2.47
S15	2.85±0.21	2.99±0.46	3.26±0.61	0.08±0.04	10.40±4.22	0.66±0.09	0.48±0.14	1.54±0.43	0.62±0.21	0.01±0.01	0.23±0.03	0.58±0.04	0.04±0.02	0.37±0.03	0.46±0.08	0.33±0.02	0.26±0.01	2.51±0.25	25.15±5.79
S16	1.83±0.70	1.78±0.61	3.33±0.27	0.03±0.01	5.17±2.91	0.41±0.06	0.40±0.07	0.73±0.28	0.79±0.87	n.d.	0.19±0.01	0.35±0.29	0.22±0.33	0.32±0.02	0.37±0.10	0.32±0.09	0.27±0.07	2.24±0.43	16.51±4.60
S17	2.24±0.72	1.78±0.31	1.71±0.88	0.04±0.04	5.71±2.15	0.45±0.13	0.53±0.18	0.71±0.27	0.80±0.38	0.02±0.04	0.21±0.04	0.55±0.14	0.05±0.02	0.36±0.04	0.37±0.10	0.34±0.08	0.28±0.04	2.48±0.58	16.17±5.32
S18	1.98±1.13	2.42±0.08	2.28±0.73	0.09±0.03	9.53±3.30	0.51±0.14	0.53±0.06	1.16±0.28	0.95±0.21	n.d.	0.19±0.04	0.47±0.03	0.04±0.01	0.32±0.04	0.34±0.07	0.25±0.01	0.20±0.02	2.14±0.11	21.26±2.38
S19	3.45±0.23	2.97±0.36	3.46±0.44	0.09±0.00	10.54±0.23	0.71±0.08	0.77±0.09	1.41±0.04	2.05±1.51	0.02±0.04	0.26±0.05	0.67±0.09	0.05±0.01	0.42±0.04	0.51±0.07	0.38±0.04	0.32±0.06	3.12±0.37	28.08±2.44
S20	2.55±0.63	2.78±0.41	1.63±0.59	0.14±0.03	11.41±0.91	0.58±0.11	0.57±0.09	1.44±0.17	0.37±0.13	0.05±0.08	0.21±0.03	0.64±0.04	0.09±0.06	0.37±0.07	0.42±0.03	0.34±0.03	0.25±0.05	2.69±0.25	23.84±0.40
L1	3.91±1.54	2.40±1.02	0.17±0.25	0.16±0.06	4.62±1.45	1.49±0.30	1.17±0.57	1.05±0.49	0.86±0.62	0.03±0.05	0.61±0.32	1.10±0.53	0.17±0.11	1.33±0.69	0.58±0.27	0.82±0.50	0.80±0.43	5.97±2.97	21.27±8.21
L2	5.25±0.72	2.56±1.11	0.28±0.43	0.13±0.10	6.75±3.59	1.80±1.06	1.31±0.26	1.21±0.34	0.69±0.25	0.01±0.02	0.57±0.22	1.16±0.46	0.16±0.09	0.97±0.47	0.62±0.28	0.70±0.35	0.76±0.36	5.69±2.25	24.93±8.33
L3	5.56±0.39	2.77±0.37	0.91±0.75	0.15±0.04	9.19±1.30	2.14±0.01	1.15±0.14	1.26±0.14	0.89±0.16	0.04±0.07	0.56±0.06	1.19±0.13	0.11±0.01	1.24±0.19	0.65±0.06	0.74±0.01	0.71±0.09	5.79±0.43	29.26±1.67
L4	8.93±0.79	3.27±0.46	n.d.	0.12±0.03	11.38±4.82	2.50±0.84	1.37±0.16	1.41±0.14	1.26±0.43	0.05±0.01	0.56±0.09	1.23±0.05	0.17±0.01	1.29±0.04	0.72±0.05	0.89±0.07	0.77±0.02	6.45±0.31	35.94±7.12
L5	9.11±1.87	2.93±0.41	0.24±0.23	0.17±0.03	8.03±3.54	2.68±1.16	1.62±0.10	1.32±0.08	1.00±0.08	0.02±0.00	0.69±0.07	1.38±0.07	0.15±0.03	1.46±0.10	0.77±0.03	1.01±0.07	0.92±0.03	7.30±0.35	33.50±4.03

L6	10.88±2.75	2.33±0.32	0.47±0.70	0.09±0.01	3.88±0.67	0.92±0.16	1.09±0.17	0.95±0.09	0.73±0.05	0.05±0.08	0.50±0.04	0.98±0.11	0.11±0.01	1.07±0.15	0.52±0.03	0.62±0.08	0.62±0.04	5.01±0.42	25.79±3.04
L7	9.69±0.78	3.13±0.33	0.07±0.05	0.31±0.09	8.53±1.54	2.16±0.38	1.26±0.16	1.43±0.18	0.71±0.14	0.12±0.06	0.26±0.39	1.25±0.01	0.10±0.08	1.33±0.04	0.72±0.05	0.91±0.15	0.80±0.01	6.38±0.20	32.80±2.28
L8	5.78±1.49	2.74±0.12	n.d.	0.30±0.01	8.86±1.04	2.84±0.83	1.50±0.09	1.23±0.08	0.76±0.24	0.04±0.00	0.62±0.13	1.15±0.06	0.16±0.06	1.23±0.12	0.63±0.04	0.83±0.04	0.79±0.08	6.29±0.34	29.47±0.62
L9	5.52±0.49	2.52±0.18	n.d.	0.19±0.07	5.75±0.07	1.43±0.23	1.36±0.16	1.23±0.05	0.77±0.02	n.d.	0.55±0.17	1.06±0.21	0.13±0.05	1.15±0.23	0.58±0.08	0.73±0.18	0.68±0.17	5.68±1.07	23.65±2.20
L10	4.79±0.27	2.74±0.13	n.d.	0.22±0.03	7.91±0.12	2.09±0.02	1.50±0.03	1.48±0.03	1.16±0.38	0.03±0.01	0.75±0.02	1.32±0.07	0.23±0.05	1.52±0.05	0.68±0.05	1.03±0.02	0.86±0.04	7.14±0.23	28.31±0.08
L11	4.63±0.69	4.60±0.06	3.40±0.54	0.20±0.04	8.88±2.40	4.01±1.71	1.65±0.10	1.48±0.09	1.04±0.11	0.03±0.01	0.81±0.09	1.59±0.17	0.24±0.02	1.74±0.22	0.89±0.13	1.06±0.15	1.07±0.10	8.24±0.77	37.32±3.89
L12	6.26±0.12	2.92±0.32	n.d.	0.14±0.01	5.28±0.29	1.47±0.07	1.54±0.08	1.12±0.04	0.94±0.14	n.d.	0.69±0.03	1.28±0.05	0.19±0.01	1.42±0.04	0.68±0.05	0.86±0.04	0.89±0.02	6.85±0.07	25.66±0.35
L13	5.56±0.37	3.22±0.22	0.65±0.48	0.17±0.03	5.45±0.98	1.45±0.31	1.60±0.15	1.20±0.14	0.91±0.22	n.d.	0.76±0.06	1.39±0.14	0.24±0.02	1.47±0.09	0.73±0.08	0.96±0.06	1.03±0.05	7.42±0.57	26.79±2.51
L14	6.05±0.28	2.97±0.16	n.d.	0.26±0.03	6.45±0.88	1.96±0.11	1.64±0.17	1.46±0.09	1.05±0.20	n.d.	0.83±0.11	1.56±0.15	0.26±0.04	1.64±0.11	0.87±0.07	1.17±0.09	1.08±0.17	8.21±0.79	29.24±1.34
L15	5.07±1.07	2.95±0.33	n.d.	0.15±0.01	5.12±0.67	1.38±0.35	1.29±0.08	1.22±0.12	0.71±0.11	n.d.	0.67±0.08	1.25±0.21	0.18±0.02	1.31±0.21	0.68±0.13	0.86±0.12	0.82±0.12	6.39±0.82	23.67±2.42
L16	5.58±0.64	2.75±0.07	n.d.	0.12±0.02	5.39±0.46	1.47±0.09	1.50±0.05	1.09±0.04	0.88±0.23	n.d.	0.65±0.01	1.27±0.06	0.17±0.01	1.40±0.04	0.66±0.04	0.83±0.03	0.87±0.04	6.70±0.18	24.64±1.33
L17	4.85±1.76	2.95±0.38	0.17±0.29	0.10±0.02	6.19±1.33	1.48±0.16	1.35±0.14	1.01±0.11	0.74±0.41	0.03±0.05	0.63±0.11	1.23±0.16	0.15±0.03	1.36±0.24	0.67±0.08	0.78±0.10	0.84±0.09	6.38±0.66	24.54±3.15
L18	5.28±0.51	3.36±0.48	0.35±0.32	0.20±0.03	7.40±0.30	2.36±0.14	1.88±0.15	1.40±0.16	0.93±0.11	n.d.	0.78±0.07	1.55±0.19	0.22±0.02	1.56±0.12	0.80±0.08	1.02±0.06	1.03±0.14	8.05±0.73	30.10±1.63
L19	6.37±0.49	2.68±0.04	n.d.	0.15±0.00	4.28±0.56	1.34±0.18	1.38±0.02	1.06±0.09	0.87±0.20	n.d.	0.63±0.02	1.21±0.08	0.18±0.01	1.32±0.12	0.66±0.06	0.85±0.04	0.84±0.08	6.45±0.41	23.84±0.69
L20	4.99±0.28	2.77±0.18	n.d.	0.20±0.02	6.80±1.01	1.77±0.25	1.38±0.09	1.30±0.09	0.39±0.33	0.08±0.01	0.74±0.04	1.43±0.03	0.21±0.04	1.54±0.07	0.82±0.02	0.98±0.03	1.01±0.05	7.36±0.27	26.40±1.01

.Note: "TEAA" means total essential amino acid; "TFAA" means total free amino acid. "R" means root; "S" means stem; and "L" means leaf. "n.d." means not detected.

Table S3. Integrate score of 20 turnip strains.

Strain	R		S		L	
	Score	Rank	Score	Rank	Score	Rank
1	-0.6943	58	-0.6148	52	0.9712	15
2	-0.6700	56	-0.4417	24	0.9455	16
3	-0.4324	23	-0.6125	51	1.1416	8
4	-0.6275	53	-0.2855	21	1.3339	1
5	-0.4735	28	-0.4792	30	1.1826	6
6	-0.4876	31	-0.6059	50	0.9054	18
7	-0.6684	55	-0.5479	41	1.3187	2
8	-0.7071	59	-0.5968	48	1.1170	10
9	-0.5573	43	-0.3365	22	1.1178	9
10	-0.4446	25	-0.4723	27	1.2196	5
11	-0.5713	45	-0.5202	36	1.2676	3
12	-0.4887	32	-0.5607	44	0.8952	19
13	-0.7184	60	-0.5779	47	1.0422	14
14	-0.5100	34	-0.4740	29	1.2591	4
15	-0.5360	39	-0.5504	42	0.9144	17
16	-0.6053	49	-0.5140	35	1.0636	12
17	-0.6667	54	-0.4930	33	1.0545	13
18	-0.5744	46	-0.5357	38	1.1796	7
19	-0.6714	57	-0.4694	26	0.8408	20
20	-0.5417	40	-0.5311	37	1.0955	11

Note: “R” means root; “S” means stem; and “L” means leaf.
